# Supplementary material for: Are carbon emissions trading and green financial instruments synergistic? -Comprehensive quantitative research based on content analysis
Source: PLoS One. 2024 Mar 7;19(3):e0298601. doi: 10.1371/journal.pone.0298601 (PMC10919601; doi:10.1371/journal.pone.0298601)
Supplement: S1 Data — (ZIP) [file pone.0298601.s001.zip › Data/地级市Z_F工作报告.zip!/░▓╗╒/║╧╖╩╩╨/└┤╫╘╣1⁄2╓┌║┼í╛┬φ┐╦╩2╛▌═°í┐_2014.docx]

各位代表:

　　现在,我代表市人民政府向大会报告工作,请予审议,并请政协委员和其他列席人员提出意见。

****一、2013年工作回顾****

　　过去的一年,我们全面贯彻落实党的十八大和十八届二中、三中全会精神,在省委省政府和市委的坚强领导下,按照“平中见奇、稳中求快、调整转型、夯实基础”的总体思路,积极应对错综复杂的宏观形势,顽强拼搏,扎实工作,保持了经济社会平稳较快发展。预计,全市生产总值4650亿元、增长11.5%;全社会固定资产投资4708亿元、增长23.1%;规模以上工业增加值1907.4亿元、增长14.5%;财政收入768.3亿元、增长10.6%,其中地方财政收入438.6亿元、增长12.6%;社会消费品零售总额1483亿元、增长15%;城镇居民人均可支配收入27980元、增长10%,农民人均纯收入10350元、增长14%;城镇登记失业率控制在4.5%以内;人口自然增长率7‰;居民消费价格涨幅2.7%;节能减排完成省控目标。

****一年来,我们主要抓好以下工作:****

　　(一)加强经济运行调节。积极落实中央和省里出台的一系列新政策,适时推出稳增长40条等政策“组合拳”,增强经济调节的前瞻性和有效性,经济发展质量和效益明显提升。努力扩大有效投入,项目建设提质提效,全年新开工项目4241个、增加229个,其中亿元以上项目947个、增加95个。鑫晟8.5代线、淀川钢板等项目建成投产,TCL冰洗基地、华南城、万达文化旅游城等开工建设,沙溪铜矿、龙桥铁矿采扩建等获得核准。促进房地产市场健康发展,商品住房交易量增价稳。完善金融机构考核办法,新增本外币贷款950亿元,实现直接融资424亿元、增长73.1%,企业债券、中期票据、短期融资券等金融工具成功运用,徽商银行赴港上市。深入推进“双百”活动,举办家博会、农产品对接会、新能源汽车展等展会170场,“万村千乡”、新网工程、“三社一会”等农村流通体系建设扎实推进,信息消费、网络购物等新兴消费加速兴起。制定促进外经贸发展政策,建立“走出去”企业孵化库,深入推进贸易便利化,实现进出口总额180亿美元。

　　(二)加快产业转型升级。完成工业技改投资1043.3亿元、增长13.8%。战略性新兴产业实现增加值533.6亿元、增长22.9%,新型显示、机器人、公共安全等列入国家区域集聚发展试点,在全国率先出台光伏产业发展和推广应用政策,国际智能语音产业园启动建设,集成电路产业取得实质性突破。出台创新型城市建设意见,获批全国首批“智慧城市”试点和国家知识产权示范城市,新认定高新技术企业143户、总数达711户,实现高新技术产业产值近4000亿元、增长18%。中科大先进技术研究院一期工程建成使用,400名工程硕士入驻,量子通信京沪干线工程正式启动,国家大科学工程未来网积极申报;清华大学公共安全研究院、合工大智能制造技术研究院等签约组建。国家级两化融合试验区建设通过验收。修编现代服务业发展规划,金融后台服务基地、物流园区、电子商务产业园、动漫及服务外包基地、特色商业街等加快建设。出台促进文化产业发展政策,推进国家级文化与科技融合示范基地、国家数字出版基地等建设,文化产业实现增加值近300亿元、增长21%。旅游总收入524亿元、增长14%,接待国内外游客近6000万人次,成为全国第三批台湾自由行城市,合肥航空公司加快筹建。

　　(三)提升城市功能品质。深入实施“1331”空间发展战略,加快编制地下空间利用、综合交通、生态体系等专项规划。推进新一轮大建设,新建、续建工程813项,完成投资281.2亿元。综合交通枢纽加快形成,新桥国际机场开通运营,高铁南站、铁路枢纽南环线、合福高铁等加快建设,合肥港综合码头二期及店埠河航道、合裕航道升级改造等工程加快实施,总里程458公里的合六、合马、合铜、合淮等7条国省公路干线改造全面开工,环巢湖旅游道路建设加快推进。轨道交通1号线全线开建、2号线进展迅速。阜阳北路与铜陵北路高架顺利竣工,全市高架路网体系基本建成;繁华大道、方兴大道中段等建成通车,龙川路、肥西路等新建改建工程进展顺利。市政公用设施不断完善,“气化合肥”步伐加快,合肥电厂6号机组及六水厂二期、七水厂一期建成使用,汽车客运西站投入运营,成功入选国家公交都市创建示范城市。深入开展“三城同创”,志愿服务实现制度化、常态化,97人荣登“中国好人榜”,居全国省会城市首位。“数字城管”建设正式启动,餐厨垃圾处理项目开工,垃圾焚烧发电一期工程投入试运营。开展市容专项整治,依法拆除非法和到期户外广告1400多处。

　　(四)推进城乡统筹发展。强化城乡融合,加快产业发展、基础设施和公共服务一体化。坚持产城一体、联动发展,四大开发区转型升级加快,城区都市产业园建设取得突破,五县(市)工业园区发展提质提效。加大支持力度,县域经济总量达1580亿元、占全市34%;县域规模以上工业增加值683.9亿元、增长15.7%。继续以“十大政策”、“十大工程”为抓手,大力支持巢湖、庐江加快发展。肥东、肥西、长丰进入全省科学发展一类县和中部百强县,肥西在全国百强县位次进一步攀升。粮食生产和畜牧水产业稳定发展,特色规模农业面积突破300万亩。改造县乡公路150公里,完成水库除险加固工程51座,建设农村饮水工程53处。

　　美好乡村建设扎实推进,136个省级中心村和930个自然村整治全面展开,49个重点示范村建设全部完成,以“三线三边”为重点的全市域环境综合整治启动实施。加大工作力度,农村扶贫开发取得新成效。

　　(五)加强生态文明建设。大力推进环巢湖综合治理,积极申报国家级环巢湖生态文明示范区。与国开行合作一期16个项目完成80%工程量,二期98个项目陆续开建,三期项目积极谋划。对74个河道全面实行“河长制”,南淝河、十五里河、兆河等中小河流治理工程加快实施,朱砖井、王小郢污水处理厂提标改造基本完成,环巢湖乡镇36座污水处理厂全部开工,建成首个藻水分离港,巢湖流域11个考核断面7个基本达标,湖区蓝藻水华面积、频次及藻密度显著减少。深入开展城乡绿化大会战,完成植树造林36万亩、城区绿化1500万平方米,大蜀山、滨湖湿地双双晋升国家森林公园,创建国家森林城市通过考核验收。午季秸秆实现重点区域全面禁烧。制定大气污染治理行动计划和应急预案,出台机动车排气污染、扬尘污染以及“黄标车”限行等管理办法,实时发布PM2.5监测数据;编制马(合)钢公司搬迁转型方案,以安徽锦邦化工各套生产装置关停为标志,化工企业全部实现退城进园。

　　(六)着力推进改革开放。国企改革不断深化,荣事达三洋与惠而浦开展战略合作,合肥赛维顺利重组,庐江矾矿破产改制稳步推进。修订促进民营经济发展条例和优化投资环境条例,出台26条支持政策,设立1亿元专项资金,推动全民创业,新登记各类市场主体近2万户、增长23%。累计132家企业参加股权和分红激励试点,33家完成,提前实现“十二五”目标。出台深化户籍管理制度改革意见。平稳实施部分乡镇行政区划调整。“营改增”扩围试点顺利推进。颁布全国首部地方法规,公共资源交易市场制度化、规范化、标准化建设实现新提升。巩固基层医改成果,9家县级公立医院综合改革全面推进,药品零差率销售落实到位。新增农业产业化龙头企业、农民合作社、家庭农场等新型农业经营主体1200多家,累计流转土地面积239万亩,庐江成为首批国家现代农业示范区农业改革与建设试点县。拓展“合肥之友”等招商平台,“百名县干大招商”等活动取得实效,欧美、东南亚、港澳台等重点区域招商成果丰硕,与央企和知名民企合作深入推进,完成招商引资2550亿元、增长22%。加强国际交流合作,荣获中美友好城市可持续发展奖。申创综合保税区进展顺利,空港经济示范区启动建设。积极促进长江中游城市集群达成武汉共识,成功承办长三角城市经济协调会第13次市长联席会。合肥经济圈一体化和扩容全面提速,滁州市整体加入。加强与皖北及皖西等县结对合作,阜阳合肥现代产业园区、寿县蜀山现代产业园区加快建设。

　　(七)加大民生保障力度。在全国率先出台基本公共服务体系规划和社会服务“1+4”政策。全面实施“33+14”项民生工程,全市民生支出484.5亿元,占财政总支出76.8%。新增城镇就业16.9万人,转移农村劳动力8.8万人。完善城乡居民养老保险政策,实现全市缴费标准、基础养老金标准、最低补贴标准“三统一”。提高城镇居民基本医疗保险保障水平,政策范围内住院费用支付比例达到70%左右。农村低保标准提高到年人均1800元以上,散居五保对象供养标准提高到年人均2200元。建立政府购买居家养老服务机制,80岁以上高龄津贴提前实现城乡全覆盖。修订廉租住房保障和管理办法,开建各类保障性安居工程4.66万套。100个老旧小区环境综合整治全面推开,惠及5.3万户居民。实施42个城中村和危旧房改造,完成房屋拆迁总面积200多万平方米。加强商贸便民体系建设,启动城区64个菜市场标准化改造,基本建成肉菜流通追溯体系。

　　(八)扎实推进社会建设。出台城市中小学布局规划和学前教育设施布局专项规划,制定推进义务教育均衡发展实施意见。高质量完成学前教育“三年行动”计划,基础教育质量稳步提升。民办学校校安工程全部达标。合肥学院应用型人才培养成为全国示范。基层卫生服务体系不断完善,新增3家全国示范社区卫生服务中心。省美术馆、科技馆、百戏城在滨湖新区开工建设,市群众文化活动中心改造工程正式动工,首批20个农民文化乐园试点全面铺开,首届全民文化周系列活动参与群众50多万人次,市民合唱团、市民交响乐团等民间文艺团体走向国际舞台。成立市社会科学院,《合肥市志》正式出版,《合肥通史》加快编纂。蜀峰湾体育公园主体工程基本建成,参赛第十二届全运会创历史最好成绩。编制社区服务体系建设规划,探索实行“大社区”等多种管理模式。深入开展安全生产大检查和食品药品等重点领域专项整治,出台加强电梯安全工作意见,实现三年烟花爆竹生产企业全行业退出目标,安全生产形势总体平稳。持续打击非法营运、非法传销,强化社会治安动态化管控,社会大局保持和谐稳定,第三次捧得“长安杯”。深入开展联合接访、党政领导干部接访下访等活动,坚持开展风险评估、矛盾调处,信访维稳取得新成效。强化利益导向机制,推进人口计生转型发展。扎实创建全国残疾人文化体育建设示范市。制定城市人防工程规划,人防民防进社区全面推进。支持民族乡村加快发展,促进宗教和睦。巩固和提升双拥模范城创建成果,创造性做好国防教育、征兵等工作,军民融合深入发展。应急管理、防震减灾、统计、气象、档案、保密、仲裁等工作取得新进展,工会、共青团、妇女儿童和关心下一代等工作实现新进步。

　　各位代表!

　　过去的一年,我们严格依法履行政府职责,自觉接受人大依法监督、政协民主监督和社会舆论监督,主动听取民主党派、工商联和社会各界意见和建议,全年办理人大代表议案和建议204件、政协提案533件。出台依法行政和法治政府建设规划纲要,制定、修改地方性法规、规章12件;大力清理行政审批事项,市本级由282项减少到152项、精简率达46%。制定深化政务公开加强政务服务实施意见,推行基本建设项目收费目录管理,开展为民服务窗口经营性收费行为专项整治,规范清理行政审批中介组织。第三次市直机关公务员转任平稳推进。首次开展政府工作创新奖评选,10个项目获奖。认真落实中央八项规定和省市相关规定,严格执行党政机关停止新建楼堂馆所和办公用房清理等要求,市直部门一般性支出一律压减5%,党政干部因公出国人数同比下降18.8%,全市性会议和文件数量大幅压缩。

　　各位代表!

　　过去的一年,我市各项工作勇于率先、争创一流,荣获一系列国家级表彰奖励,部分经济指标在全国26个省会城市中跻身或接近“十强”,实现了本届政府的良好开局。这是省委省政府和市委坚强领导的结果,是市人大、市政协及社会各界大力支持的结果,是全市广大干部群众团结奋斗的结果。在此,我谨代表市人民政府,向全市广大工人、农民、知识分子、干部、驻肥解放军指战员、武警官兵和政法干警,向各民主党派、工商联、人民团体和社会各界人士,向所有关心支持合肥改革开放与现代化建设的海内外朋友,表示衷心的感谢,并致以崇高的敬意!

　　在总结成绩的同时,我们也清醒地看到存在的问题:发展不足、发展不优、发展不平衡的矛盾仍然较为突出,生产总值、城镇居民人均可支配收入两项指标增幅没有达到预期目标;经济下行压力加大,土地、资金、用工等要素供求矛盾凸显;在城市管理、征地拆迁、信访维稳等领域,矛盾纠纷增多,管理难度加大;巢湖综合治理任务艰巨,大气污染防治形势严峻,资源环境约束趋紧;思想解放程度和改革创新力度与形势任务要求尚有差距,政府职能转变、效能提升和工作落实仍存在薄弱环节,“四风”问题还不同程度存在。我们一定加倍努力工作,采取切实有效措施解决好这些问题。
